# Reliability of power output, maximal rate of capillary blood lactate accumulation, and phosphagen contribution time following 15‐s sprint cycling in amateur cyclists

**DOI:** 10.14814/phy2.16086

**Published:** 2024-05-23

**Authors:** Benedikt Meixner, Valentin Nusser, Karsten Koehler, Mattice Sablain, Jan Boone, Billy Sperlich

**Affiliations:** ^1^ Integrative and Experimental Exercise Science & Training Julius‐Maximilians‐Universität Würzburg Würzburg Germany; ^2^ Department of Sport Science and Sport Friedrich‐Alexander‐Universität Erlangen‐Nürnberg Erlangen Germany; ^3^ Iq‐Move PG Lochmann & Fraunberger Erlangen Germany; ^4^ TUM School of Medicine and Health, Department of Health and Sport Science Technical University of Munich Munich Germany; ^5^ Department of Movement and Sports Sciences Ghent University Ghent Belgium

**Keywords:** alactic, ATP, energy, high‐intensity, metabolism, muscle

## Abstract

Based on Mader's mathematical model, the rate of capillary blood lactate concentration (νLa_max_) following intense exercise is thought to reflect the maximal glycolytic rate. We aimed to investigate the reliability of important variables of Mader's model (i.e. power output, lactate accumulation, predominant phosphagen contribution time frames (t_P__Cr_)) and resulting νLa_max_ values derived during and after a 15‐s cycling sprint. Fifty cyclists performed a 15‐s all‐out sprint test on a Cyclus2 ergometer three times. The first sprint test was considered a familiarization trial. Capillary blood was sampled before and every minute (for 8 min) after the sprint to determine νLa_max_. Test–retest analysis between T2 and T3 revealed excellent reliability for power output (P_mean_ and P_peak_; ICC = 0.99, 0.99), ∆La and νLa_max_ with t_PCr_ of 3.5 s (ICC = 0.91, 0.91). νLa_max_ calculated with t_PCr_ = t_P__peak_ (ICC = 0.87) and t_P__Cr_ = t_Ppeak–3.5%_ (ICC = 0.79) revealed good reliability. t_Ppeak_ and t_Ppeak–3.5%_ revealed only poor and moderate reliability (ICC = 0.41, 0.52). Power output and ∆La are reliable parameters in the context of this test. Depending on t_PCr_, reliability of νLa_max_ varies considerably with t_P__Cr_ of 3.5 s showing excellent reliability. We recommend standardization of this type of testing especially t_P__Cr_.

## 1 INTRODUCTION

Many test procedures and physiological variables exist to judge the aerobic and anaerobic metabolic components involved in high‐intensity cycling. In recent years the post‐exercise level of capillary blood lactate accumulation has received increasing interest in order to calculate the glycolytic capacity in cycling (Adam et al., 2015; Dunst et al., 2023; Quittmann, Abel, et al., 2021; Quittmann, Schwarz, et al., 2021; Yang et al., 2023), hand‐cycling (Quittmann, Abel, et al., 2021; Quittmann et al., 2018) and other endurance sports including running (Quittmann et al., 2022; Quittmann, Appelhans, et al., 2020; Quittmann, Schwarz, et al., 2021), swimming (Mavroudi et al., 2023; Teixeira et al., 2022) and rowing (Schünemann et al., 2023). In this regard the peak accumulation of capillary blood lactate within 10 min after a 15‐s all‐out sprint (often abbreviated as νLa_max_) has been widely discussed among athletes and within the coaching community as a feasible indicator to calculate the glycolytic capacity during sprint cycling (note: here νLa_max_ is preferred over “ċLa_max_” due to its established recognition) (Heck & Schulz, 2002; Mader, 2003; Mader & Heck, 1986; Quittmann et al., 2022).

Short maximal efforts (such as 15‐s sprint cycling) rely largely on the phosphocreatine pathway as well as glycolysis to regenerate ATP rapidly (Heck & Schulz, 2002; Nevill et al., 1994; Yang et al., 2023). Therefore, the ability to produce lactate quickly is considered one important predictor of sprint performance. νLa_max_ serves as one necessary variable for a metabolic model developed by Mader (Mader, 2003; Mader & Heck, 1986). Within Mader's model, the νLa_max_ is thought to indirectly estimate the maximal activity of phosphofructokinase as the bottleneck of glycolysis. PFK, the principal regulator of glycolysis, is activated by the presence of ADP, AMP, and inorganic phosphate, and is inhibited by ATP, H+ ions, and citric acid (Mader, 2003).Consequently, the rate of lactate accumulation in the blood serves as an indirect indicator of glycolytic activity. This rationale underpins the standardized duration of 15 seconds for the test, which is designed to ensure maximal glycolytic activation through the accumulation of ADP and AMP, while minimizing inhibition by H+ ions.

As one consequence the elevated rate of νLa_max_ in Mader's model is indicative of increased reliance of glycolytic energy production.

νLa_max_ is commonly calculated as proposed by Mader (Heck & Schulz, 2002; Mader, 1984).

$$\mathrm{vL}a_{\max}=\frac{∆\mathrm{La}}{t_{\text{glycolytic}}}=\frac{La_{\text{peakpost}}-La_{\mathrm{pre}}}{t_{\text{test}}-t_{\mathrm{PCr}}}$$

With *La_peakpost_* as the highest post‐exercise capillary blood lactate value, La_pre_ the capillary blood lactate value measured immediately before sprinting, *t*_test_ = the sprint time (which in a 15‐s cycle sprint is set to 15 s) and *t_PCr_* as the assumed time of predominant phosphagen contribution of the test. As Mader's original model is rooted in enzymatic rates, the inclusion of a time variable in the denominator (Mader & Heck, 1986) becomes essential. This addition is imperative due to the time‐dependent nature of enzyme‐catalyzed reactions and serves to represent the duration during which glycolysis plays a role in energy production.

Based on the formula, the value of the denominator is heavily influenced by the t_PCr_ and its determination. Unfortunately, several methods to determine t_PCr_ exist including the time from sprint start to peak power (P_peak_) (Manunzio et al., 2016) or the time from sprint start until reaching P_peak_ and subsequently declining by 3.5% (Adam et al., 2015; Quittmann, Schwarz, et al., 2021) prohibiting the comparison of results between studies. In situations where the requisite sampling rates for determining specific measures are not available, a practical duration of 3.5 seconds can be adopted, following the recommendations provided by Heck and Schulz (Heck & Schulz, 2002). Interestingly, although t_PCr_ is a very important element in the formula its determination has not been discussed much (Dunst et al., 2023; Yang et al., 2023) and no thorough reliability investigation of the different t_PCr_ exists so far. The process of calculating νLa_max_, which hinges on a foundation of physiological data, is subject to typical variability inherent in such measurements. This variability underscores the importance of ensuring high reliability in these calculations. This reliability is not just a technical concern but is fundamental for making accurate and informed interpretation where νLa_max_ is applied. Therefore, rigorous validation and verification of these data are essential for the integrity and efficacy of decision‐making processes based on νLa_max_ metrics.

So far a limited number of studies including a low sample sizes (*n* = 17–23) assessed the reliability of νLa_max_ in amateur running (*n* = 18); (Quittmann, Schwarz, et al., 2021), hand‐cycling (*n* = 18) (Quittmann, Abel, et al., 2021), rowing (*n* = 17) (Held et al., 2023) and cycling (*n* = 18–23; sport students) (Adam et al., 2015; Quittmann, Abel, et al., 2021; Quittmann, Schwarz, et al., 2021) but these included different sprint durations, amount of involved active muscle mass (i.e. upper and lower body muscle) and different t_test_‐ t_PCr_ time portion for νLa_max_ calculation. Based on the available information, the question arises how reliable the νLa_max_ quantification is in experienced amateur cyclists when employing different t_PCr_.

Therefore, the goal of the present experiment was to investigate the reliability of important variables of Mader's model derived during and after 15‐s sprint cycling performance (i.e. peak and mean power output, post‐exercise capillary blood lactate measurements and different t_PCr_ measurements). We hypothesize that the corresponding parameters related to νLa_max_‐testing display robust reliability allowing for their further application in science and practice.

## 2 METHODS

### 2.1 Participants

A cohort of *n* = 50 (*n* = 30 male, *n* = 20 female) experienced cyclists with more than 3 years of regular cycling exercise (>2 sessions per week) were recruited for this study. All participants were experienced in road cycling with clipless pedals and cycled regularly as exercise. Prior to the study, the participants were informed of the protocol and gave their written informed consent to participate. All procedures were approved by the ethical committee of Exercise Science & Training of the Faculty of Human Sciences (EV2024/1–1004) and conducted in accordance with the Declaration of Helsinki (Harriss & Atkinson, 2009). Participants characteristics are given in Table 1.

**TABLE 1:** Mean ± SD age, body stature, selected anthropometric data and peak oxygen uptake of participants.

Variable	All (*n* = 50)
Age (years)	31.2 ± 7.8
Height (cm)	177.5 ± 9.3
Body mass (kg)	71.7 ± 12.4
Body fat (%)	13.9 ± 4.8
Fat‐free mass (kg)	61.9 ± 11.5
Maximum oxygen uptake (mL/kg/min)	55.4 ± 7.6

### 2.2 Experimental design

**FIGURE 1:**
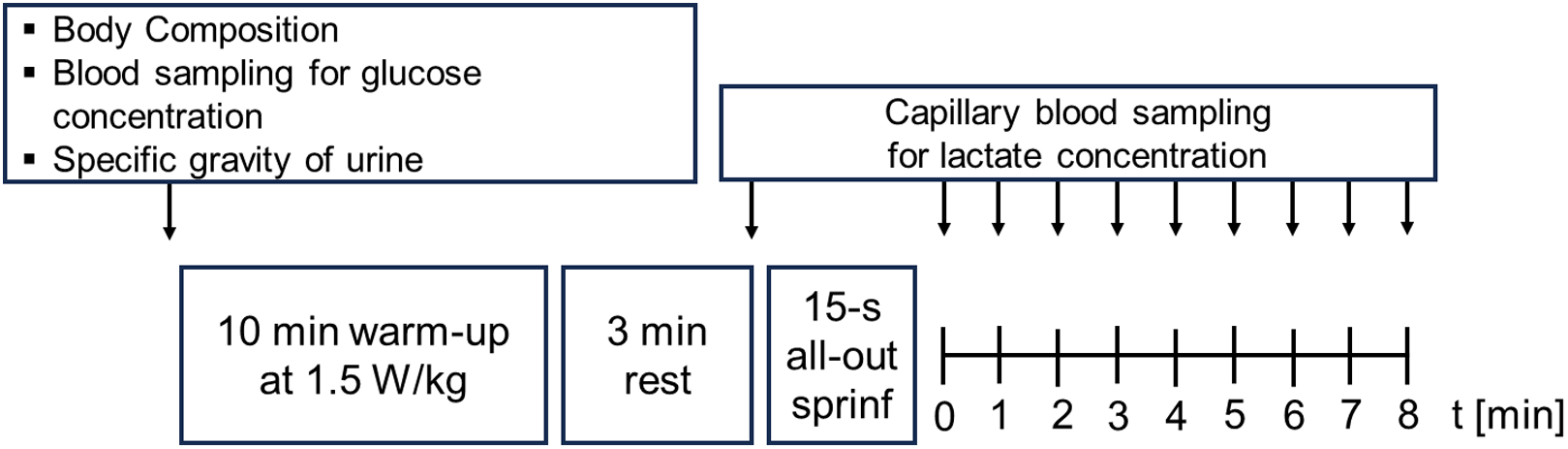
Illustration of the time line of all study procedures.

Three visits to the laboratory were required which were at least 48 h apart and completed within a period of 2 weeks. Figure 1 illustrates the timeline and all testing procedure for each visit.

All participants were instructed to keep a nutrition diary and to repeat their usual diet for each visit within the 24 h before each visit (Jeacocke & Burke, 2010). Additionally, all were instructed to stay adequately hydrated, to eat a carbohydrate‐rich meal (i.e. a banana and a jam sandwich) no less than 3 h before each visit and to refrain from caffeine consumption. Each participant received 35 g of a carbohydrate mixture (IsoFast, DextroEnergy, Krefeld, Germany) dissolved in 500 mL of water to drink ad libitum during warm‐up and recovery periods. All participants provided a urine sample in a cup first when visiting the laboratory. Hydration status was then promptly tested via urine specific gravity analysis employing a dipstick (One step 10, DFI Co., Gyeongsangnam‐do, South Korea).

During the first visit, body composition that is, fat‐free mass (FFM) of all participants were measured employing eight‐electrode impedance analysis (InBody 720, Biospace, Des Moines, Iowa, USA).

All cycle sprints were conducted on a Cyclus2 ergometer (RBM, Leipzig, Germany) and their own personal road bike. The Cyclus2 is an electromagnetically braked ergometer and measures power with an accuracy error of 2% according to the manufacturer. All cyclists used their own shoes and pedals for all tests. For all three visits, all cyclists warmed up for 10 min cycling at 1.5 W/kg body mass and resting for 3 min (Quittmann, Schwarz, et al., 2020).

The all‐out cycle sprint was performed in a seated position utilizing the large chainring (if applicable) of the participant's bike and the 15‐tooth cog of the ergometer. Recording of the test started with cadence of >30 RPM. The ergometer software was set to isokinetic mode and 130 RPM (Adam et al., 2015; Nitzsche et al., 2018; Quittmann, Schwarz, et al., 2020).

Capillary blood samples of the left earlobe were sampled twice during the resting period, after the warm‐up and once directly after the sprint as well as every minute for 9 min after the 15‐s cycle sprint. Lactate concentration was measured amperometric‐enzymatically employing Biosen C‐Line (EKF Diagnostics, Barleben, Germany).

#### 2.2.1 Analysis of t_P__Cr_

Sprint power data were processed with Winlaktat (6, mesics, Münster, Germany) to determine values for t_Ppeak_ and t_Ppeak–3.5%_. t_Ppeak_ was calculated as the time from start of the test until peak power output was reached (Manunzio et al., 2016). t_Ppeak–3.5%_ was calculated as the time from the start of the test until peak power was reached and subsequently decreased by 3.5% or more (Quittmann, Schwarz, et al., 2021). Existing measures were employed to preclude the introduction of an additional time frame, with t_Ppeak–3.5%_ being utilized to reflect the accuracy error of an early version of the SRM ergometer (Yang et al., 2023). Additionally, a fixed t_PCr_ of 3.5 s was used for calculations. This time frame represents the mean of Heck and Schulz's assumptions of t_PCr_ = 3 s for a test duration of 10s and t_PCr_ = 4 s for a test duration of 20s and is a time frame commonly used in absence of adequate sampling rates for determination of t_Ppeak_ and t_Ppeak–3.5%_.

### 2.3 Statistical analyses

Raw data was processed using Microsoft Excel. Statistical analyses (mean, standard deviations, and 95% confidence intervals) were computed with GraphPad Prism (10, Boston, MA, USA). Data normality for all measured variables (P_peak_, P_mean,_ ∆La, FFM, body mass) was assessed using the Shapiro–Wilk test and visual inspection, without requiring further transformation.

Since T1 represented a familiarization trial we excluded this data for further reliability testing.

Intraclass correlations were calculated using JASP 0.18.1 (ICC, Model: Alpha, two‐way random; Type: absolute agreement, single measures) for all relevant measures of the cycle sprint test (P_peak_, P_mean,_ ∆La, t_Ppeak_ and t_Ppeak–3.5%_) and subsequent calculations of νLa_max_ (with t_PCr_ = 3.5 s, t_Ppeak_ and t_Ppeak–3.5%_). (Koo & Li, 2016; Shrout & Fleiss, 1979). Interpretation was based on the 95% confidence interval of the ICC estimate. Values less than 0.5, between 0.5 and 0.75, between 0.75 and 0.9, and greater than 0.90 are indicative of poor, moderate, good, and excellent reliability, respectively (Koo & Li, 2016).Standard error of measurement (SEM) was calculated from the square root of the mean square error term in a repeated measures ANOVA according to the method of Stratford and Goldsmith and Eliasziw et al (Atkinson & Nevill, 1998; Eliasziw et al., 1994; Stratford & Goldsmith, 1997) for measures of the cycle sprint test (P_peak_, P_mean_, ∆La, t_Ppeak_ and t_Ppeak–3.5%_) and calculations of νLa_max_ (with t_PCr_ = 3.5 s, t_Ppeak_ and t_Ppeak–3.5%_). Coefficient of Variation was calculated as the ratio of SEM to the grand mean of T2 and T3 (Weir & Vincent, 2020).

Bland–Altman plots and limits of agreement were calculated using the difference between methods compared to the average for the varying calculation methods of νLa_max_ (with t_PCr_ = 3.5 s, t_Ppeak_ and t_Ppeak–3.5%_) for T2 and T3 (Martin Bland & Altman, 1986). 95% Limits of agreement and bias was calculated according to the method of Bland–Altmann for P_peak_, P_mean_, ∆La, t_Ppeak_, t_Ppeak–3.5%_ and all νLa_max_ calculations in T2 and T3 (Martin Bland & Altman, 1986).

## 3 RESULTS

All mean ± SD data for all _variables_ assessed during the 15‐s cycle sprints are summarized in Table 2.

**TABLE 2:** All variables (Mean ± SD) obtained during and after the relevant (T2, T3) 15‐s cycle sprints used for analysis.

Variable	T2 (second visit)	T3 (third visit)
νLa_max_ (3.5 s) [mmol/l/s]	0.55 ± 0.14	0.54 ± 0.13
νLa_max_ (t_Ppeak_) [mmol/l/s]	0.50 ± 0.11	0.49 ± 0.13
νLa_max_ (t_Ppeak–3.5%_) [mmol/l/s]	0.56 ± 0.13	0.53 ± 0.14
P_peak_ (W)	970 ± 272	974 ± 283
P_mean_ (W)	758 ± 195	752 ± 194
Total Work (J)	11,373 ± 2927	11,284 ± 2912
P_mean_/FFM [W/kg]	12.16 ± 1.47	12.06 ± 1.49
La_Pre_ [mmol/L]	0.90 ± 0.26	0.80 ± 0.22
La_maxpost_ [mmol/L]	7.18 ± 1.63	7.03 ± 1.81
∆La [mmol/l]	6.28 ± 1.57	6.23 ± 1.79
t_Ppeak_ [s]	2.45 ± 0.87	2.29 ± 0.81
t_Ppeak–3.5%_ [s]	3.77 ± 1.35	3.40 ± 1.30
Urine specific gravity (g/mL)	1.020 ± 0.33	1.016 ± 0.013
Blood glucose [mmol/L]	5.5 ± 0.7	5.4 ± 0.5

Abbreviations: νLa_max_ (3.5 s) = νLa_max_ calculated with t_PCr_ = 3.5 s; νLa_max_ (t_Ppeak_) = νLa_max_ calculated with t_PCr_ = t_Ppeak_; νLa_max_ (t_Ppeak–3.5%_) = νLa_max_ calculated with t_PCr_ = t_Ppeak–3.5%_; P_peak_ = peak power during the sprint test; P_mean_ = average power during the sprint test; P_mean_/FFM = average power during the sprint test normalized to fat‐free mass; La_pre_ = blood lactate concentration before the sprint test; La_maxpost_ = maximal blood lactate concentration in 8 minutes after the sprint test; ∆La = difference between La_pre_ and La_maxpost_; t_Ppeak_ = time until P_peak_ was reached; t_Ppeak–3.5%_ = time until P_peak–3.5%_ was reached.

### 3.1 Reliability calculation T2, T3

**FIGURE 2:**
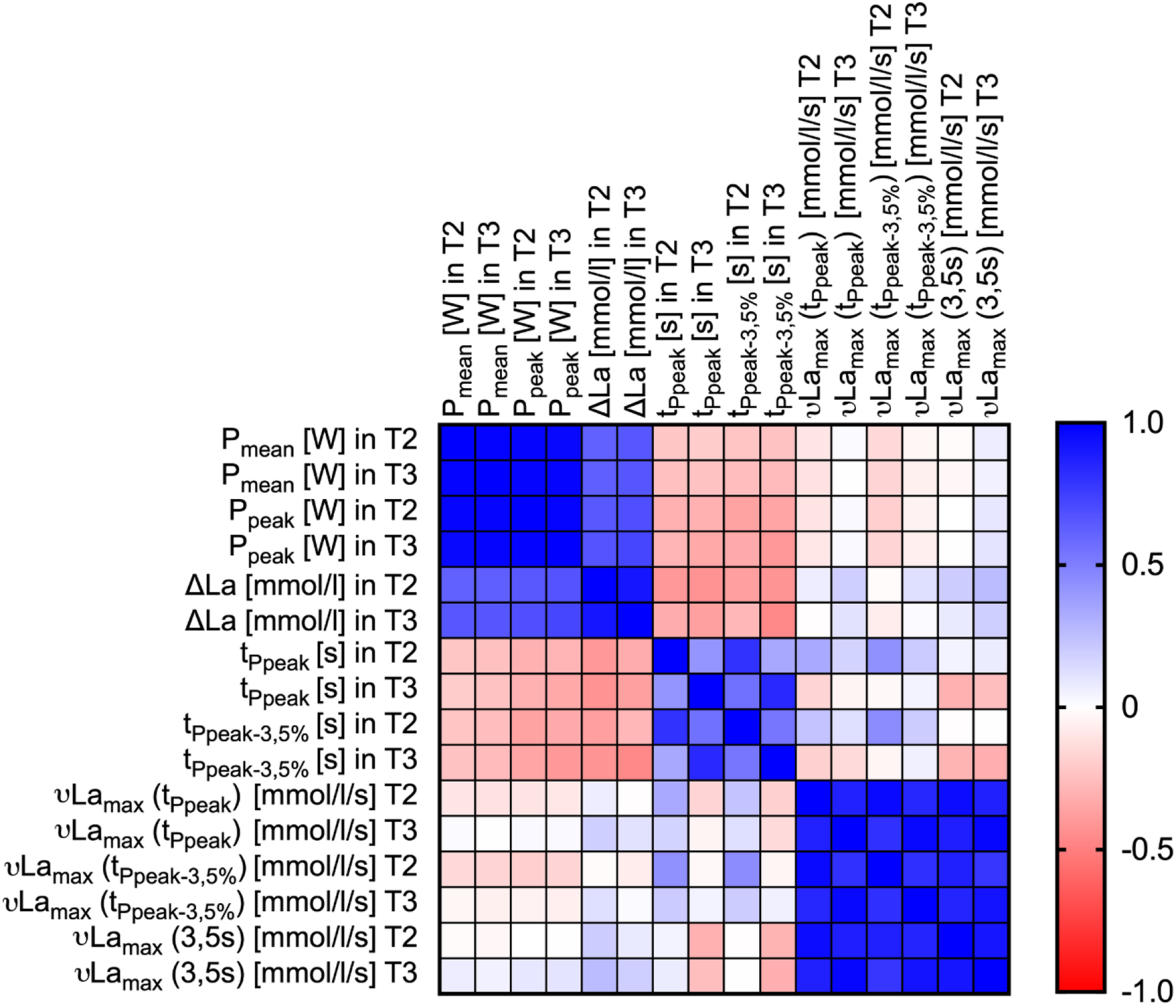
Correlation matrix of all relevant variables during the 15‐s sprint, P_mean_ = mean power output, P_peak_ = peak power output, ∆La = difference between La_pre_ and La_maxpost_, t_Ppeak_ = time until P_peak_ was reached, t_Ppeak–3.5%_ = time until P_peak–3.5%_ was reached, νLa_max_ (t_Ppeak_) = νLa_max_ calculated with t_PCr_ = t_Ppeak_, νLa_max_ (t_Ppeak–3.5%_) = νLa_max_ calculated with t_PCr_ = t_Ppeak–3.5%_, νLa_max_ (3.5 s) = νLa_max_ calculated with t_PCr_ = 3.5 s.

The correlation matrix of all variables T2 vs. T3 are illustrated in Figure 2. Reliability measures are summarized in Table 3.

**TABLE 3:** Intraclass correlation coefficient (ICC) (95% confidence interval) and standard error of measurement (SEM) calculated for T2 and T3.

	ICC	SEM	CoV	Bias	95% LoA
νLa_max_ (3.5 s) mmol/l/s	0.911	0.02	3.1%	0.00	−0.13 to 0.12
	(0.849–0.949)				
νLa_max_ (t_Ppeak_) [mmol/l/s]	0.866	0.06	12.1%	−0.01	−0.15 to 0.13
	(0.777–0.922)				
νLa_max_ (t_Ppeak–3.5%_) [mmol/l/s]	0.794	0.15	26.4%	−0.03	−0.23 to 0.17
	(0.660–0.879)				
**∆**La [mmol/l]	0.911 (0.849–0.949)	0.22	3.6%	−0.05	−1.44 to 1.35
P_peak_ [W]	0.986	23	2.4%	4.6	−87.4 to 96.5
	(0.975–0.992)				
P_mean_ [W]	0.986	30	3.6%	−5.9	−69.9 to 58.0
	(0.975–0.992)				
t_Ppeak_ [s]	0.414	0.71	30.0%	−0.14	−1.91 to 1.62
	(0.158–0.618)				
t_Ppeak–3.5%_ [s]	0.520	1.83	51.0%	−0.37	−2.87 to 2.14
	(0.289–0.695)				

Abbreviations: νLa_max_ (3.5 s) = νLa_max_ calculated with t_PCr_ = 3.5 s; νLa_max_ (t_Ppeak_) = νLa_max_ calculated with t_PCr_ = t_Ppeak_; νLa_max_ (t_Ppeak–3.5%_) = νLa_max_ calculated with t_PCr_ = t_Ppeak–3.5%_; ∆La = difference between La_pre_ and La_maxpost_; P_peak_ = peak power during the sprint test; P_mean_ = average power during the sprint test; t_Ppeak_ = time until P_peak_ was reached; t_Ppeak–3.5%_ = time until P_peak–3.5%_ was reached.

**FIGURE 3:**
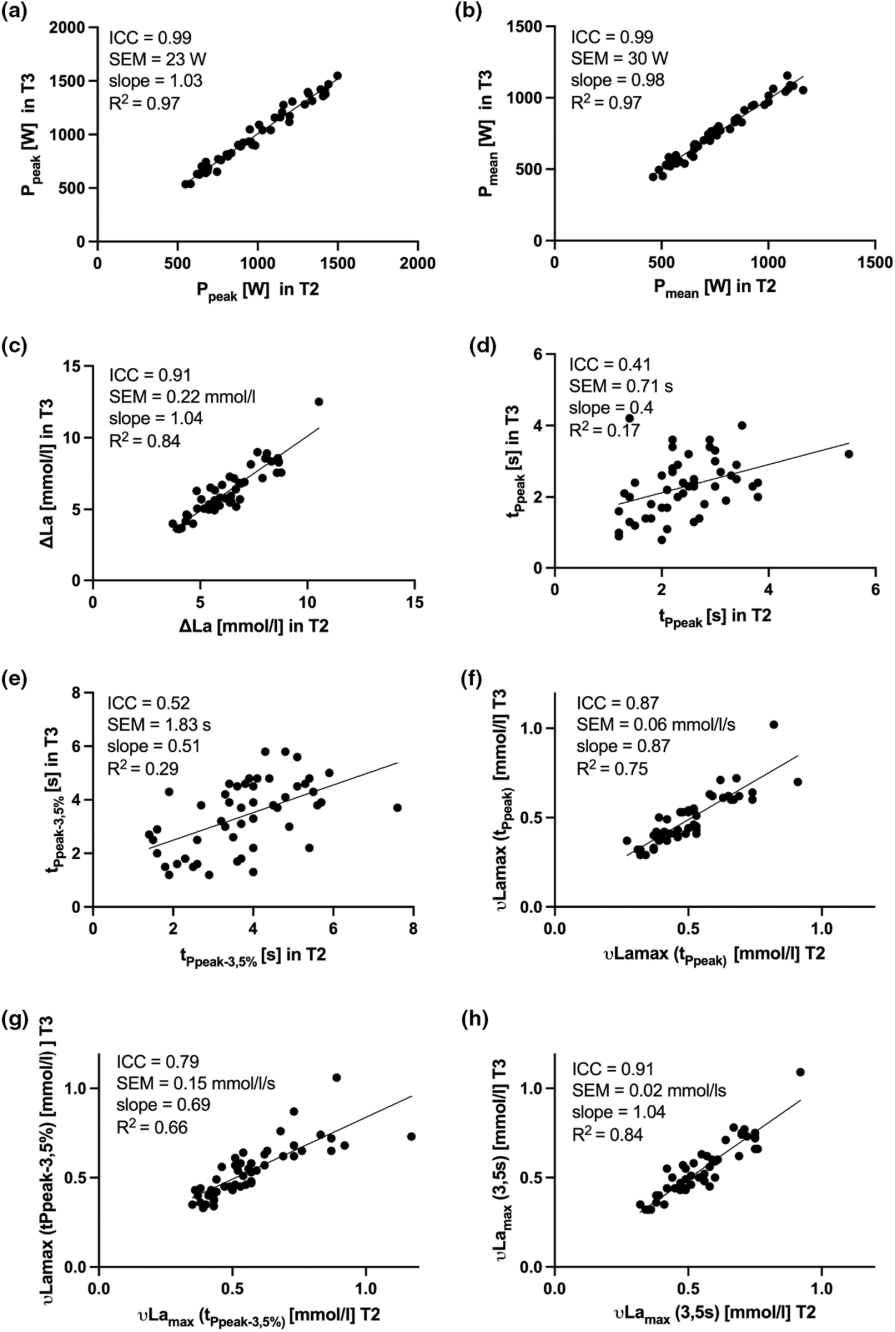
Correlation for T2 and T3 in (a) P_peak_, (b) P_peak_, (c) ∆La, (d) t_Ppeak_, (E) t_Ppeak–3.5%_, (f) *νLa*_*max*_ (t_Ppeak_), (g) *νLa*_*max*_ (t_Ppeak–3.5%_), (h) *νLa*_*max*_ (3.5 s).

Figure 3a–h illustrates the data of each variable of interest (P_peak_, P_mean_, **∆**La, t_Ppeak,_ t_Ppeak–3.5%_, νLa_max_ (3.5 s), νLa_max_ (t_Ppeak_), νLa_max_ (t_Ppeak–3.5%_)) and cyclist between T2 and T3.

As main result P_mean_ (ICC = 0.99, CoV = 3.9%) and P_Peak_ (ICC = 0.99, CoV = 2.4%) revealed excellent retest reliability while **∆**La showed good to excellent reliability (ICC = 0.91, CoV = 3.6%). Across trials 2 and 3, νLa_max_ revealed excellent, good and good reliability respectively for the different t_PCr_ calculation methods: t_PCr_ = 3.5 s (ICC = 0.91, CoV = 3.1%), t_PCr_ = t_Ppeak_ (ICC = 0.87, CoV = 12.1%), t_PCr_ = t_peak–3.5%_ (ICC = 0.79, CoV = 26.4%).

**FIGURE 4:**
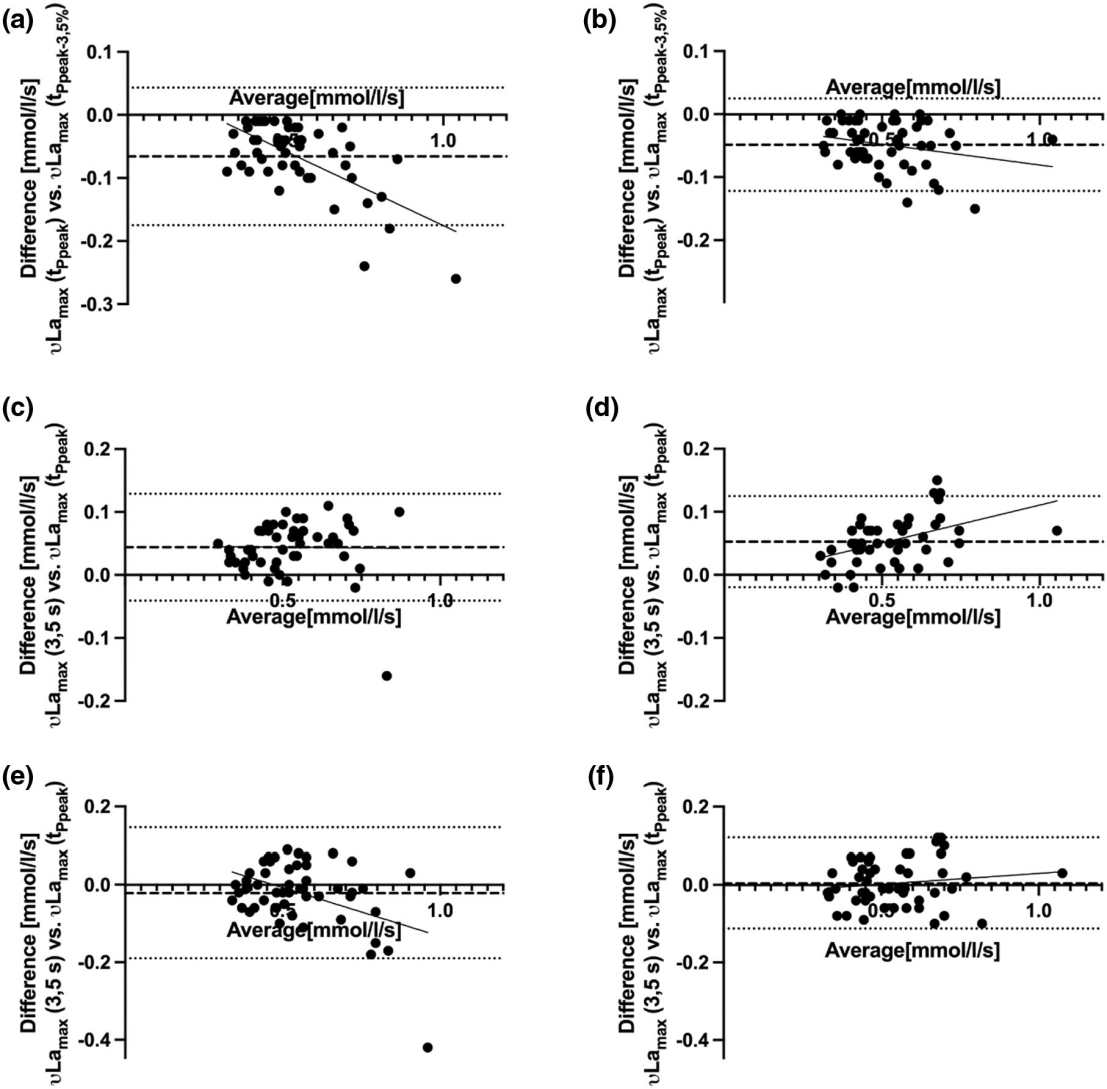
Bland–Altman analysis for *νLa*_*max*_ (t_Ppeak_) and *νLa*_*max*_ (t_Ppeak–3.5%_) in T2 (a) and T3 (b), *νLa*_*max*_ (3,5 s) and *νLa*_*max*_ (t_Ppeak_) in T2 (c) and T3 (d), *νLa*_*max*_ (3,5 s) and *νLa*_*max*_ (t_Ppeak–3.5%_) in T2 (e) and T3 (f).

Figure 4a–f illustrates the Bland–Altman plots of νLamax values corresponding to the different calculation methods for tPCr νLamax (3.5 s), νLa_max_ (t_Ppeak_), νLa_max_ (t_Ppeak–3.5%_) in T2 and T3 respectively.

### 3.2 T_PCr_ variables

**FIGURE 5:**
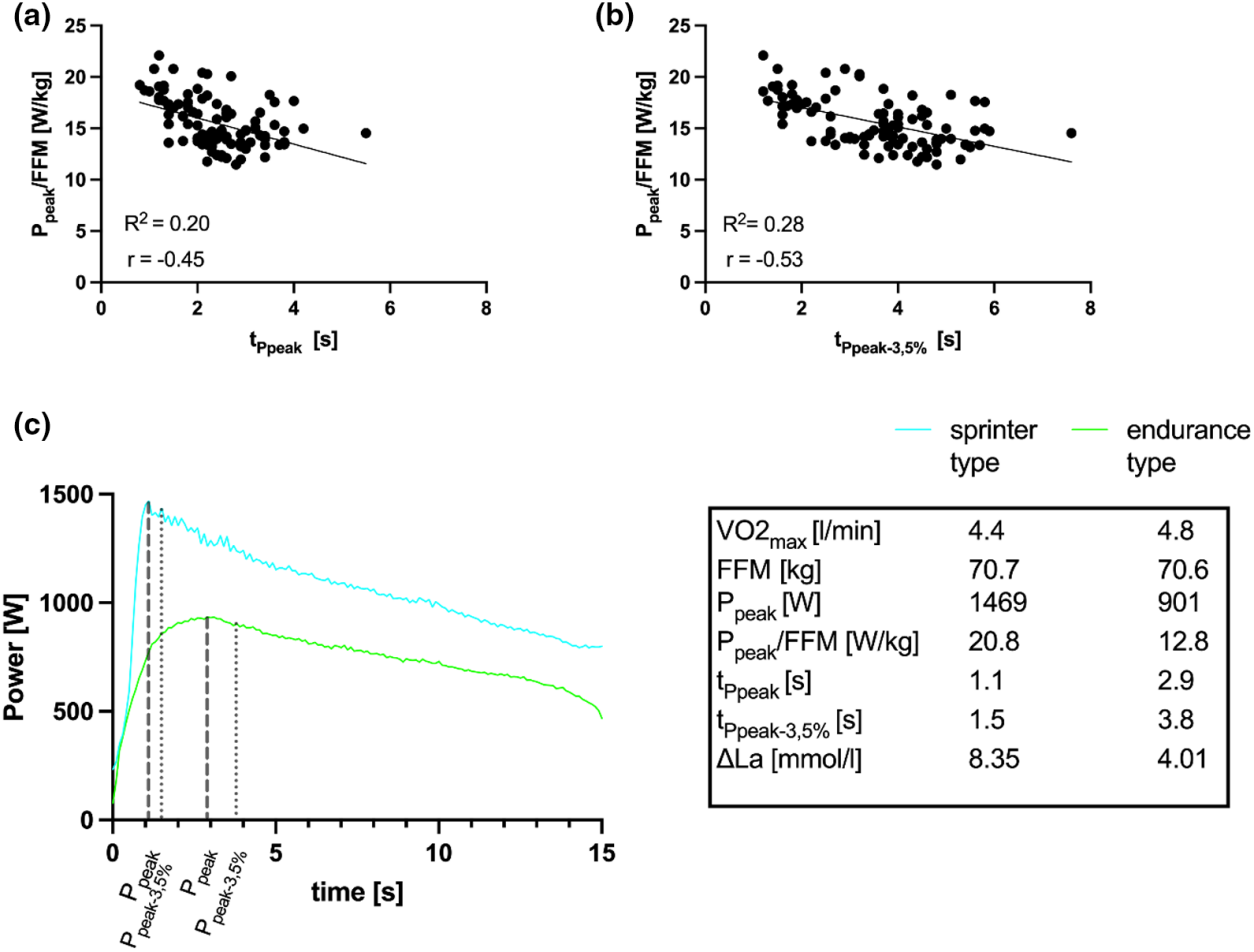
Relation between P_peak_/FFM and t_Ppeak_ (a) and t_Ppeak–3.5%_ (b), (c) example of sprint power data over 15 s, P_peak_, t_Ppeak_, P_peak–3.5%_ and t_Ppeak–3.5%_ for two male cyclists of similar stature and body mass for sprinter type versus endurance type (see legend for individual data).

Measurements for t_Ppeak_ and t_Ppeak–3.5%_ revealed poor to moderate test–retest reliability (ICC = 0.39; =0.52, CoV = 30.0%; =51.0%, Figure 3d,e). Additionally, a weak relationship appeared between increased P_peak_ and decreased t_Ppeak_ as well as t_Ppeak–3.5%_ values (Figure 5a,b). Exemplary data is displayed in Figure 5c.

## 4 DISCUSSION

The main findings of the present investigation are:

- Test‐restest reliability for peak and mean power output was excellent
- **∆**La and subsequently, νLa_max_ with t_PCr_ of 3.5 s showed excellent reliability
- T_PCr_ measures (t_Ppeak_ and t_Ppeak–3.5%_) display poor to moderate reliability resulting in decreased reliability of the νLa_max_ calculation employing these measures. Overall reliability of the equations was moderate to good.

### 4.1 Reliability assessment

The present investigation revealed excellent test–retest reliability of P_peak_ and P_mean_ and excellent reliability for νLa_max_ with t_PCr_ of 3.5 s between T2 and T3.

The high test–retest reliability for maximal blood lactate accumulation in sprint cycling has been also demonstrated in earlier studies assessing reliability of νLa_max_ with t_Ppeak–3.5%_ (Adam et al., 2015; Quittmann, Schwarz, et al., 2021). Based on our and the recent data, we may conclude that νLa_max_ with a fixed time frame for predominant phosphagen contribution constitutes a variable with high test‐restest reliability in connection with 15‐s sprint cycling.

Given that directly measuring intramuscular lactate production in sprint cycling is presently impractical, recent and older studies have explored various metabolic pathways through simulations employing Mader's model. (Hauser et al., 2014; Ji et al., 2021; Mader & Heck, 1986). However, these simulations are highly dependent on νLa_max_ and its calculation, i.e., the denominator and the t_PCr_ component. The present findings showed that depending on which t_PCr_ was employed the νLa_max_ calculation differed extensively. Based on the findings of our study, we recommend a standardized application of t_PCr_, specifically in our case t_PCr_ of 3.5 s because this variable revealed the highest degree of reliability. We assume that the integration of different time frames of t_PCr_ may at least partly explain the discrepancy between data obtained from the simulations compared to actual performance testing (Hauser et al., 2014; Ji et al., 2021).

As mentioned before, the determination of “predominant phosphagen contribution time” is an important factor in the calculation of the maximal rate of lactate production in Mader's model. As an example, in a 15 s sprint test, a change of 1 s in the time span of predominant phosphagen contribution could influence the resulting νLa_max_ up to 26% (Dunst et al., 2023). This represents a hypothetical time parameter that significantly impacts the denominator in the νLa_max_ calculation. However, the application of an “predominant phosphagen contribution time span” seems disputable: depending on the transport and diffusion of lactate from the muscle into and within the blood stream an time without lactate accumulation may occur when employing and interpreting the kinetics of capillary blood lactate measurements in connection with sprint cycling. However, when considering the glycolytic activity within the working muscle cell it seems questionable whether an time span with no blood lactate accumulation exists (Brooks, 2018; Chung et al., 1998; Dunst et al., 2023).

Based on the present findings, the most reliable data was observed in connection with fixed time span, i.e. t_PCr_ of 3.5 s. However, as elucidated before, the question remains whether this time should be measured at all. We are well aware that other quantification methods of an t_PCr_ exists including the determination by the power curve when P_peak_ is reached (Quittmann, Abel, et al., 2021), by the power curve with P_peak–3.5%_ (Quittmann, Schwarz, et al., 2021), by individual determination of the force/velocity profile (Dunst et al., 2023) and by relative contribution of glycolysis while measuring phosphagen and oxidative contribution with breath‐by‐breath measurement (Yang et al., 2023). Nevertheless, to the best of our knowledge, none of these methods underwent rigorous reliability assessment in connection with sprint cycling. Noteworthy, the t_PCr_ measures including P_peak_ as a marker are influenced by the initial inertia resistance of the ergometer: in case the initial resistance is set too low for the cyclist, P_peak_ is reached rapidly vice versa when the initial resistance is too high, power outputs will increase even during increasing muscular fatigue (Dunst et al., 2023). Our results show that the determination of the t_PCr_ by the power curve (t_Peak_) is not a reliable measure for determination of νLa_max_. However, this finding may partly be caused by the employed ergometer and its settings.

Adding to that, Figure 5c shows lower t_PCr_ in the cyclists with higher P_peak_/FFM. This is in line with the findings by Dunst et al. (Dunst et al., 2023). The decreased t_PCr_ (in the cyclists with lower P_peak_/FFM) increases the denominator (t_test_‐ t_PCr_) of Mader's equation, resulting in lower νLa_max_ values compared to the cyclist with greater P_peak_/FFM and shorter t_PCr_. Differences between athletes in power output and ∆La may therefore appear reduced when expressed as νLa_max_. Therefore, the use of a fixed t_PCr_ for the determination of νLa_max_ seems to reveal physiological differences more consistently.

### 4.2 Strength and limitations

We consider our sample size (*n* = 50) a strength of this study. Comparable studies in the field provided a sample size of 18–23 (Hauser et al., 2014; Quittmann, Schwarz, et al., 2021; Yang et al., 2023). Further all participants considered themselves as true cyclists or triathletes, not as runners or physical education students being tested as unexperienced cyclist once or twice on a bike.

The measure of νLa_max_ is used in endurance cycling and triathlon and therefore dependent on the accompanying body angles and acclimatization to the position and contact points on the bike. Therefore, the use of the participants own bike is necessitated which was the case in the present study in contrast to other studies (Adam et al., 2015).

Determination of t_PCr_ is controversial and crucial for calculation of νLa_max_. The time frame of t_Ppeak–3.5%_ is rooted in a reduction of power output by 1%, factoring in the accuracy error of the SRM of 2.5%. In our setup, considering the error accuracy of 2% of the Cyclus2 ergometer, t_Ppeak–3.5%_ could have been used. However, considering the ongoing discussion in this matter (Dunst et al., 2023), we avoided creating an additional time frame for t_PCr_. Determination of t_PCr_ by the force/velocity profile as used by Dunst et al. (Dunst et al., 2023) necessitates additional testing and the method was not available at the time of data collection. Moreover, the approach proposed by Yang et al. (Yang et al., 2023) has not undergone reliability testing and could potentially gain from the integration of a force/velocity profile analysis.

While we ensured control over hydration status through specific gravity of urine testing, body composition was assessed using bioelectrical impedance analysis due to the unavailability of more advanced methods for body composition.

### 4.3 Conclusion

In conclusion, the reliability of νLa_max_ varies considerably when employing different time frames for predominant phosphagen contribution with t_PCr_ of 3.5 s showing the highest and good level of reliability. Changes in physiology might be best represented using a fixed t_PCr_ or absolute values in ∆La.

## FUNDING INFORMATION

Authors state no funding involved.

## CONFLICT OF INTEREST STATEMENT

Authors state no conflict of interest.

## Data Availability

The data that support the findings of this study are available on reasonable request from the corresponding author. The data are not publicly available due to privacy or ethical restrictions.
